# High Seroprevalence for Spotted Fever Group Rickettsiae, Is Associated with Higher Temperatures and Rural Environment in Mbeya Region, Southwestern Tanzania

**DOI:** 10.1371/journal.pntd.0003626

**Published:** 2015-04-07

**Authors:** Norbert Heinrich, Tatjana Dill, Gerhard Dobler, Petra Clowes, Inge Kroidl, Mandy Starke, Nyanda Elias Ntinginya, Leonard Maboko, Thomas Löscher, Michael Hoelscher, Elmar Saathoff

**Affiliations:** 1 Division of Infectious Diseases and Tropical Medicine, Medical Center of the University of Munich (LMU), Munich, Germany; 2 German Center for Infection Research (DZIF), Munich partner site, Munich, Germany; 3 Bundeswehr Institute of Microbiology, Munich, Germany; DZIF German Center for Infection Research, Munich partner site, Munich, Germany; 4 German Partnership Program for Excellence in Biological and Health Security, Munich, Germany; 5 NIMR-Mbeya Medical Research Centre, Mbeya, Tanzania; University of Tennessee, UNITED STATES

## Abstract

**Background:**

Rickettsioses are endemic in sub-Sahara Africa. Burden of disease, risk factors and transmission are hitherto sparsely described.

**Methods:**

From the EMINI (Evaluating and Monitoring the Impact of New Interventions) population cohort, we randomly selected 1,228 persons above the age of 5 years from the nine participating communities in Mbeya region, Southwestern Tanzania, stratified by age, altitude of residence and ownership of domestic mammals, to conduct a cross-sectional seroprevalence study in. The aim was to estimate the seroprevalence of IgG antibodies against Spotted Fever Group (SFG) rickettsiae and to assess socioeconomic and environmental risk factors. Serology (indirect immunofluorescence) was performed at a dilution of 1:64.

**Results:**

SFG-seropositivity in the cohort was found to be 67.9% (range among nine sites: 42.8–91.4%). Multivariable analysis revealed an association with age (prevalence ratio, PR per 10 years: 1.08; 95% CI 1.06–1.10), warmer temperatures (PR per °C: 1.38; 1.11–1.71), male gender (PR 1.08; 1.00–1.16), and low population density (PR per 1.000 persons/km²increase 0.96; 0.94–0.99). At higher elevations, higher cattle density was associated with higher seroprevalence.

**Conclusion:**

SFG rickettsial infection seems to be common in the more rural population of Mbeya Region. Spread seems to be further limited by temperature and higher elevation. Examination of the contribution of SFG to febrile illnesses seems warranted in a prospective study to estimate the disease burden in the population. This will also allow determination of the causative pathogens.

## Author Summary

We report a high seroprevalence for antibodies against Spotted Fever Group (SFG) rickettsiae in Southwestern Tanzania, a group of bacteria that is mostly transmitted by ectoparasites such as fleas, lice, mites, and ticks. Serum samples from 1.228 persons were selected, and 67.9% were positive indicating past infection. Seropositivity was clearly associated with warmer temperature, low population density and elevations below 1,500 m. These infections may have been caused by *Rickettsia africae*, which is transmitted to humans by a cattle tick, and was found in similar settings in Africa. To investigate further on the significance of SFG rickettsioses on disease burden in the area, investigations in acutely febrile patients should be conducted.

## Introduction

Tick-borne rickettsiae were first detected in the early 20th century, and have frequently been reported from all over the world including the Mediterranean and sub-Sahara African countries. Until recently all spotted fever cases in Africa were suspected to be Mediterranean Spotted Fever (MSF), caused by the obligatory intracellular bacterium *Rickettsia conorii*. In 1996, Kelly et al. identified the new species *R*. *africae* as the pathogenic agent of African Tick Bite Fever (ATBF) and as part of the Spotted Fever Group (SFG) rickettsiae [1]. Therefore Pijper’s suggestion from 1936 to differentiate between spotted fevers with a more and less severe prognosis [2] could be fortified, with ATBF falling into the latter group [3].

In travel medicine, tick-borne rickettsioses are regarded as the second most frequently diagnosed tropical disease entity in febrile patients returning from rural sub-Sahara Africa [4,5]. High rates of antibody seropositivity against SFG rickettsiae have been reported for the populations of many African countries such as Angola, Burkina Faso, the Central African Republic, the Ivory Coast, Congo, Mali [6], Kenya [7], Mauritania [8], Zambia [9], Zimbabwe [10,11] and most recently Senegal [12]. In northern Tanzania, 8% of acutely febrile hospitalized patients were serologically diagnosed with SFG rickettsia infections [13]. In contrast, a study on febrile pediatric outpatients <10 years from western and central Tanzania found a rickettsial cause in only 1% (10 of 1005); with six children diagnosed with typhus group, and four with a SFG rickettsial infection by serology. While the epidemiological importance of SFG rickettsioses in Africa is increasingly recognised, only few data are available on the distribution of SFG rickettsial species, the burden and the severity of disease, geographic localisation and on risk factors for acquiring infection, which could probably help in understanding the differences between the two Tanzanian cohorts mentioned. Consequently, misdiagnosis and mistreatment are frequent and preventive measures are rare.

The typical clinical triad in rickettsiosis consists of a maculopapular rash, fever and an eschar, but the occurrence of symptoms and the prognosis vary between the different types of spotted fevers [12]. Pathogens like *R*. *conorii* and *R*. *massiliae* seem to be related to a more severe disease, while ATBF, which is caused by *R*. *africae*, and presents with fever and often multiple eschars, is a benign disease. Vectors and reservoir hosts differ between SFG species. While *R*. *africae* is transmitted mostly by the cattle ticks *Amblyomma hebraeum* or *variegatum*, [12], *R*. *conorii* and *R*. *massiliae* are found predominantly in the brown dog tick *Rhipicephalus sanguineus* and disease caused by those agents is more likely to be contracted in urban areas [2].

### Methods

The following description of methods and population were already included in previously published reports with serostudies on different infectious agents; except for the serological method employed here [14–16].

### Ethics

Both EMINI and this sub-study were approved by Mbeya Medical Research and Ethics Committee, and the Tanzanian national Medical Research Coordinating Committee. Each EMINI participant had provided written informed consent before enrolment. Parents consented for participation of their minor children. Data and samples were anonymized using an alphanumeric code. Linkage to personal identifying information was only possible via a key database to which only the head of data management at the Tanzanian site had access.

### Study population

The EMINI study was a population-based survey with longitudinal follow up, created for providing the basis to **E**valuate and **M**onitor the **I**mpact of **N**ew **I**nterventions in the Mbeya Region of Southwestern Tanzania.

Data and samples for this study were collected between June 2007 and June 2008 during the second annual survey of the EMINI cohort study. Before the start of EMINI, a census of the complete population had been conducted in nine geographically distinct sites of the Mbeya Region of Southwestern Tanzania, which had been selected to represent a wide variety of environmental and infrastructural settings including urban and rural sites, different proximity to main roads and elevation above sea-level. During the census we collected basic information regarding the households and their inhabitants, and recorded all household positions, using handheld GPS receivers. Ten percent of the census households and all their inhabitants were chosen by geographically stratified random selection to participate in the 5-year longitudinal EMINI cohort study, resulting in a representative sample of the population in the nine study sites. Every year, each participating household was visited to conduct structured interviews and to collect blood and other specimen from all household members. Blood samples were cryopreserved after cells were separated from serum.

For this sub-study, we stratified the 17,872 participants, who had provided a blood sample in the second EMINI survey, by their age, altitude of residence and ownership of domestic mammals, to be able to assess factors of interest that had been identified in the literature but might have been underrepresented in the general population. We employed disproportionate random sampling with equal participant numbers for each stratum to identify samples from 1228 participants above the age of 5 years to be tested for IgG antibodies against Spotted Fever Group (SFG) rickettsiae using *R*.*conorii* as surrogate antigen.

### Socio-economic status

During the annual EMINI visits, we conducted interviews with the head of each household regarding the socio-economical and infrastructural setting in and around the household. With this information we constructed an SES score to characterize the socio-economic situation of each household, employing a modification of a method originally proposed by Filmer and Pritchett that uses principal component analysis and has been widely applied to assess wealth and poverty in developing countries [17–19]. The score included the following information: Availability of different items in the household (clock or watch, radio, television, mobile telephone, refrigerator, hand cart, bicycle, motor cycle, car, savings account); sources of energy and drinking water; materials used to build the main house; number of persons per room in the household and availability and type of latrine used.

### Environmental data

Population- and livestock-densities were calculated using data and household positions collected during the initial population census. Elevation data were retrieved from the NASA Shuttle Radar Topography Mission (SRTM) global digital elevation model, version 2.1 [20,21]. Land surface temperature (LST) and vegetation cover (EVI = enhanced vegetation index) data for the years 2003 through 2008 were retrieved from NASA’s Moderate-resolution Imaging Spectroradiometer (MODIS) mission which “are distributed by the Land Processes Distributed Active Archive Center (LP DAAC), located at the U.S. Geological Survey (USGS) Earth Resources Observation and Science (EROS) Center (lpdaac.usgs.gov).” [22]. These data were used to produce long-term averages of day and night LST and EVI. Population-, household-, and livestock-densities, LST, EVI, and elevation data were averaged for a buffer area within 1000 meter radius around each household in order to characterize the ecological situation around the household. This approach was preferred to using the respective spot values at the household position, because spot data are more prone to random error than averages for a wider area.

### Serology

Serum samples were tested for antibodies against SFG-Rickettsia by indirect immunofluorescence (IIFA) with a commercially available test (Rickettsia conorii Spot IF, Fuller Laboratories, Fullerton, U.S.A.). Serum samples were used at a dilution of 1:64, and developed using a polyclonal rabbit anti-human IgG immunoglobin labelled with fluorescein isothyocyanate. All slides were independently examined by two experienced laboratory workers. In case of discrepancies results were discussed and slides compared to positive controls until agreement was reached.

Commercial negative and positive controls were used in each test. Fluorescence of the rickettsiae with an intracellular distribution and intensity pattern similar to the positive control was considered as a positive reaction. Titres of 64 or higher were considered sero-reactive and further titration of the sera was not done.

### Data analysis

Stata statistics software (version 13, StataCorp., College Station, TX, USA) was used for all statistical analyses, and Manifold System 8.0 Professional Edition (Manifold Net Ltd, Carson City, NV) was used for processing of geographical data and to produce maps. In order to identify possible risk factors for SFG IgG positivity, we analysed seropositivity as the binary outcome in uni- and multi-variable Poisson regression models with robust (or Huber-White) variance estimates adjusted for household clustering [23,24]. Initial uni-variable models for all factors that we deemed as possibly related to SFG infection were used to identify variables with a uni-variable p-value < = 0.1 for further multi-variable evaluation. The following variables were included into multi-variable evaluation without consideration of their p-values: age and gender (as basic confounders), and the two cattle related variables (because of the assumed role of cattle in the transmission of SFG). Continuous variables were categorized into quintiles and both, representations of the data examined. If possible, we used the continuous variable, but if the categorical representation of the variable showed strong evidence of a nonlinear association with SFG IgG positivity (e.g. a strongly concave or convex association), the categorical version was used. Stepwise backward and forward regression, the Akaike and Bayes information criterion and various assessments of model-fit were used to identify the final multi-variable model, where only variables with a multi-variable p-value <0.1 were retained. If variables were strongly collinear (which was common in the environmental variables, e.g. ambient temperature, rainfall, elevation, slope, vegetation density etc.) as witnessed by a variance inflation factor > = 5, the variable that provided the best model fit was retained in the final multi-variable model. Study site was not included into the final model because it was strongly collinear with the environmental variables. All ecological variables in the final model were tested for two-way interaction, and the interaction terms included into the model, if a significant interaction was present.

## Results

**Fig 1:**
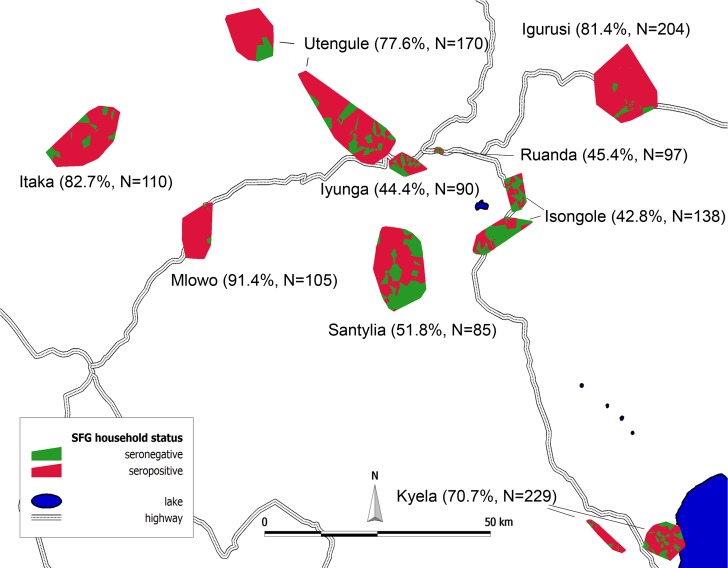
Location of households with SFG IgG-positive participants displayed as Voronoi polygons, with every polygon representing one household.

Of the 1,228 analysed sera, 67.9% overall were positive for SFG IgG. Seropositivity varied considerably between the nine different sites (Table 1 and Fig 1).

**Table 1:** Characteristics of the Study Population and their places of residence.

	Igurusi (N = 204)	Mlowo (N = 105)	Santylia (N = 85)	Isongole (N = 138)	Itaka (N = 110)	Utengule (N = 170)	Ruanda (N = 97)	Kyela (N = 229)	Iyunga (N = 90)	Total (N = 1228)
SFG seroprevalence	81.4%	91.4%	51.8%	42.8%	82.7%	77.6%	45.4%	70.7%	44.4%	67.9%
Age^a^	38.3	33.5	36.2	37.0	28.5	35.6	33.9	29.5	36.7	33.9
IQR	21.8 to 53.3	17.2 to 46.1	20.4 to 51.2	15.9 to 56.4	13.9 to 45.0	18.6 to 54.3	16.5 to 51.9	15.5 to 50.8	17.6 to 53.9	17.1 to 52.0
Socio-Economic-Status Score^a^	-0.19	0.29	-0.49	-0.42	-0.22	-0.20	1.17	-0.55	0.50	-0.14
IQR	-0.65 to 0.48	-0.03 to 0.80	-0.81 to -0.04	-0.77 to 0.18	-0.64 to 0.26	-0.59 to 0.36	0.71 to 1.79	-1.02 to 0.06	0.14 to 1.86	-0.64 to 0.52
Population Density (/km^2^) ^a^	1,541	4,022	205	626	216	217	11,788	471	1.513	461
IQR	284 to 2,173	223 to 4780	139 to 291	337 to 888	150 to 278	95 to 286	11,547 to 12,069	344 to 731	1,070 to 2,187	223 to 1,956
Cattle (/km^2^) ^a^	163	265	33	24	42	37	252	169	70	81
IQR	54 to 205	76 to 287	21 to 44	11 to 72	30 to 73	27 to 50	244 to 258	140 to 186	54 to 81	37 to 187
Distance to main road (km) ^a^	0.4	0.8	16.6	0.3	29.0	5.0	0.2	3.9	0.7	2.2
IQR	0.2 to 0.9	0.5 to 1.1	14.7 to 18.8	0.13 to 0.95	26.9 to 30.9	3.4 to 10.9	0.12 to 0.32	2.5 to 6.0	0.3 to 1.2	0.4 to 6.2
Elevation (m) ^a^	1,193	1,580	2,018	2,009	1,509	1,346	1,714	487	1,604	1,491
IQR	1,155 to 1,205	1,575 to 1,585	1,985 to 2,068	1,892 to 2,223	1,477 to 1,554	1,274 to 1,449	1,711 to 1,728	483 to 514	1,591 to 1,619	1,157 to 1,710
max. EVI ^a^	0.50	0.45	0.54	0.49	0.42	0.53	0.26	0.53	0.47	0.50
IQR	0.49 to 0.51	0.43 to 0.47	0.52 to 0.54	0.48 to 0.53	0.41 to 0.44	0.52 to 0.55	0.25 to 0.28	0.50 to 0.56	0.44 to 0.49	0.45 to 0.53
Avg. Land Surface Temp. (°C) ^a^	34.6	33.6	29.9	28.0	34.3	33.0	31.9	32.3	33.5	33.3
IQR	34.2 to 35.7	33.3 to 33.7	29.2 to 30.8	26.1 to 29.0	34.1 to 34.6	31.1 to 34.0	31.6 to 32.6	30.8 to 33.5	33.3 to 33.7	30.8 to 34.1

a: medians for respective sites

IQR = interquartile range

### Socio-economic risk factors

**Fig 2:**
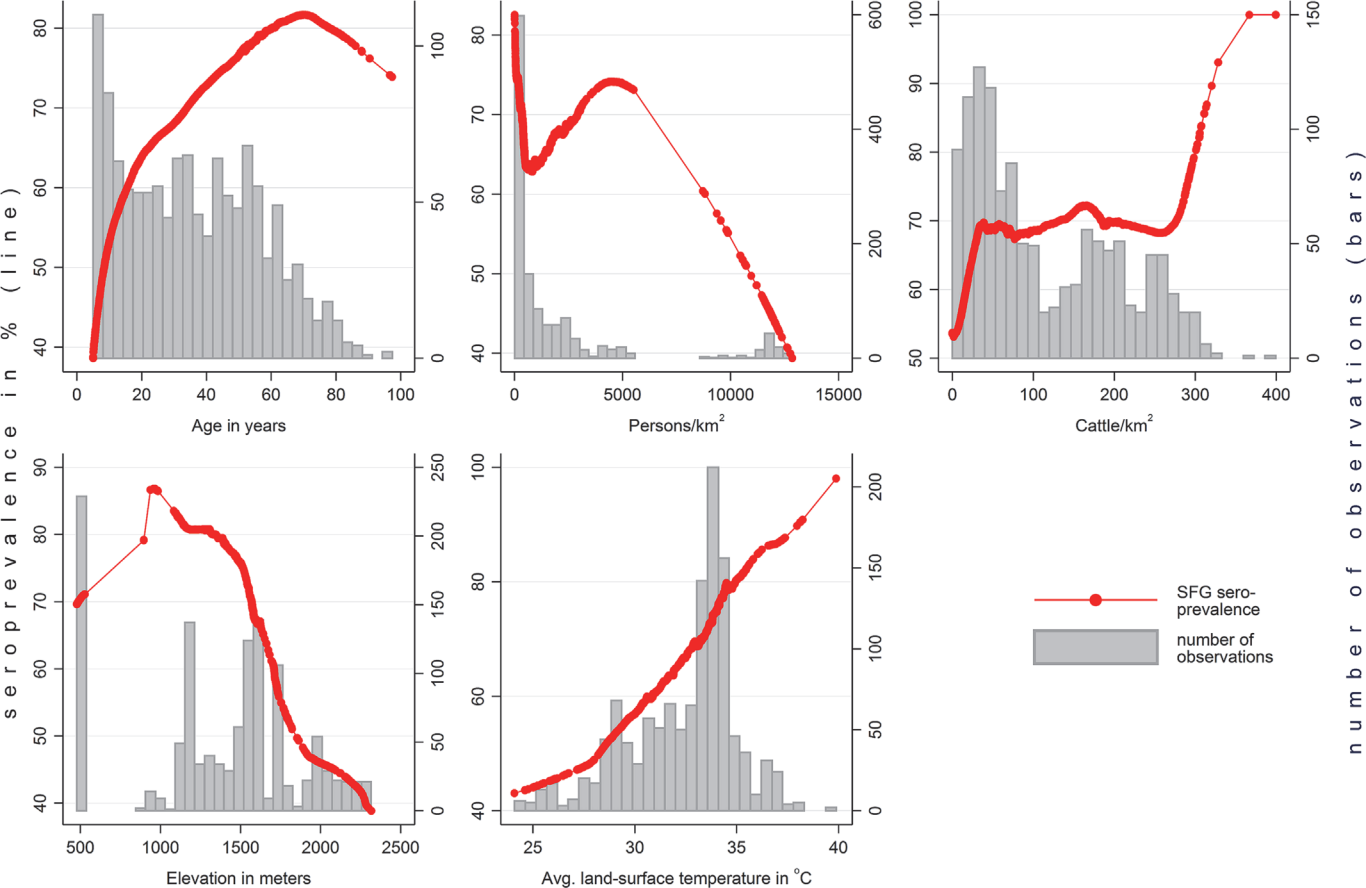
Lowess smoothed plots of SFG-seroposivitivity over age, population density, cattle density, temperature and elevation. Thin lines represent extrapolations, i.e. areas without data.

Seropositivity increased with age (Table 2 and Fig 2), with a prevalence of 43.6% in children between 5 and 8 years of age, the youngest age group examined.

**Table 2:** Socio-demographic and environmental factors and their association with Spotted Fever Group Rickettsial Seropositivity (N = 1228).

			uni-variable					Multi-variable		
Covariate	stratum	N^a^	% pos.^b^	PR^c^		95% CI^d^	p-val	PR^c^	95% CI^d^	p-val
**Age**										
	per 10 years	1228	67.9	1.07		(1.05 to 1.09)	<0.001	1.08	(1.06 to 1.10)	<0.001
**Gender**										
	female	674	66.2	1		-	-	1	-	-
	male	546	70.0	1.06		(0.98 to 1.14)	0.160	1.08	(1.00 to 1.16)	0.040
	miss.	8	75.00	1.13		(0.76 to 1.70)	0.544	1.15	(0.82 to 1.63)	0.414
**Persons/km** ^**2**^										
	per 1000 persons	1228	67.9	0.97		(0.96 to 0.99)	0.001	0.93	(0.90 to 0.96)	<0.001
**Average Land Surface Temperature**										
	per unit	1228	67.9	1.93		(1.63 to 2.28)	<0.001	1.31	(1.05 to 1.64)	0.016
**Elevation (meters)**										
	479-	245	71.8	1		-	-	1	-	-
	974-	246	80.9	1.13		(1.02 to 1.25)	0.022	1.00	(0.80 to 1.26)	0.971
	1291-	245	81.6	1.14		(1.03 to 1.26)	0.013	0.95	(0.76 to 1.19)	0.656
	1578-	246	58.9	0.82		(0.72 to 0.94)	0.004	0.66	(0.51 to 0.87)	0.003
	> = 1725	246	46.3	0.65		(0.55 to 0.76)	<0.001	0.59	(0.43 to 0.80)	0.001
**Interaction of Elevation with Cattle/km** ^**2**^										
	479-							1	-	-
	974-							1.05	(0.91 to 1.21)	0.516
	1291-							1.21	(1.04 to 1.39)	0.011
	1578-							1.35	(1.14 to 1.59)	<0.001
	> = 1725							1.41	(1.09 to 1.82)	0.008
**Cattle/km** ^**2**^										
	per 100 animals	1228	67.9	1.03		(0.99 to 1.08)	0.145	1.00	(0.88 to 1.13)	0.995
**SES score** ^e^										
	per unit	1228	67.9	0.95		(0.91 to 0.99)	0.013	*This and the following variables were not included into the multi-variable model due to lack of multi-variable significance*		
**Number of cattle owned**										
	per animal	1228	67.9	1.01		(1.01 to 1.02)	<0.001			
**Number of goats owned**										
	per animal	1228	67.9	1.01		(0.99 to 1.03)	0.215			
**Distance to nearest highway**										
	per km	1228	67.9	1.01		(1.00 to 1.01)	<0.001			
**Rainfall (in mm)**										
	101.4-	235	63.4	1		-	-			
	114.6-	227	70.5	1.11		(0.97 to 1.27)	0.114			
	123.9-	274	84.3	1.33		(1.19 to 1.49)	<0.001			
	147.2-	246	55.3	0.87		(0.75 to 1.02)	0.079			
	192.4-	246	64.2	1.01		(0.88 to 1.16)	0.855			
**Enhanced vegetation index (max.)**										
	per 0.1 units	1228	67.9	1.09		(1.03 to 1.15)	0.004			
**Slope**										
	per degree	1228	67.9	0.98		(0.96 to 1.00)	0.015			

Results of poisson regression analysis with robust variance estimates adjusted for household clustering.

a: Number of participants in each stratum (stratified variables only)

b: percent sero-positive in stratum

c: Prevalence ratio

d: 95% confidence interval

e: Socio-Economic-status according to socio economic score

Male participants were significantly more often seropositive than females (multi-variable analysis: prevalence ratio (PR) = 1.08, 95% confidence interval (CI) = 1.00 to 1.16). Seropositivity was higher in areas with low population density (PR = 0.93 per 1000/km^2^, CI = 0.90 to 0.96). An association of SFG IgG with socio-economic status, which was significant in uni-variable analysis, was rendered non-significant in the multi-variable model.

### Ecological risk factors

Seropositivity was strongly associated with the annual average land surface temperature during the day (PR = 1.31 per °C, CI = 1.05 to 1.64).

Elevation is associated with SFG IgG in uni-variable and multi-variable models, but the relation was non-linear, with a significant decline above 1,578 meters.

Since elevation and rainfall could not be included into the final model simultaneously due to collinearity, rainfall was excluded since elevation was the better predictor of infection.

Cattle density showed a non-significant trend for positive association in uni-variable analysis. Multi-variable interaction analysis with elevation showed cattle density to be positively associated with seropositivity only in higher elevation strata above 1291 m. The association with number of cattle owned was rendered non-significant in the multi-variable model, suggesting that the local density of animals is more important than ownership. Other suspected risk factors seemed to have no significant impact such as the possession of goats (PR in uni-variable analysis 1.01, 95% CI 0.99–1.03, p = 0.215), pigs (PR 0.97, 95% CI 0.88–1.07; p = 0.064), chicken (PR 1.04, 95% CI 0.95–1.14, p = 0.353), or dogs (PR 1.02, 95% CI 0.91–1.14, p = 0.750).

## Discussion

Our data show that lifetime risk of SFG infection in the study area is very high, with seroprevalences up to 80% in higher age strata. Many infections already seem to occur in early childhood below the age of 5 years.

Several socio-economic and environmental factors seem to play a role in infection. Higher temperatures as one of the stronger risk factor are biologically plausible, since those seem to increase host-seeking behaviour in many ticks [25,26], among other influences on tick breeding and survival.

Further, our analyses find associations with lower population density in multi-variable analysis, thus incidence seems higher in rural communities. Population itself may not be the only driving factor here: in our setting this variable and vegetation density are inversely collinear; thus one of both factors usually drops out of multi-variable analysis, with limited certainty on which factor is a better predictor of SFG IgG seropositivity [14]. Vegetation is a proxy for water and humidity, so a denser vegetation could mean a more humid environment; dryness reportedly affects tick host-seeking behaviour in several tick species [25,26]. Comparing these data to our published analysis of typhus group rickettsial (TGR) antibodies, it is evident that TGR and SFG seropositivity occur in different geographical patterns, with higher prevalence in areas with sparse vegetation; or dense population for TGR [14]. TGR in our area probably rely on a rat reservoir, which is probably more abundant in cities. The likely vector is *Xenopsylla cheopsis*, the rat flea, which may be less dependent on elevation, outer temperature and humidity than the SFG vector due to more continuous proximity to its vertebrate host.

The non-linear association of SFG seropositivity with elevation could be due to two independent effects. High elevations with their colder and rougher conditions are likely to negatively affect the tick vector carrying the pathogen. The lowest elevation stratum (below 974 m) with lower seroprevalence, consists of one site, Kyela. This site has abundant collections of surface water, and may thus be unsuitable to tick development despite it’ s favourable warm and moist climate. Contrarily to SFG, the mosquito-borne diseases like Rift Valley fever or Alphavirus show the highest seroprevalences in this site, which is probably due the availability of surface water [15,16].

The predominant SFG species remains to be established. The association of seropositivity with cattle (which is evident in higher elevation strata above 974 m) allows speculations whether a cattle parasite could be involved as vector, and/or cattle could play a role as a reservoir host for the pathogen. A countrywide survey of cattle ticks in Tanzania by others identified *Amblyomma variegatum*, the vector of *R*. *africae*, as the predominant cattle tick [27]. Further, high rates of SFG seropositivity were described in cattle from Zimbabwe, a country where *A*. *variegatum* is also endemic [11,28].

Preliminary results from our group, examining ticks collected from the study area by PCR, found 6 out of 10 *A*. *variegatum* ticks were positive for *R*. *africae*. *R*.*massiliae* was found in 2 out of 7 *Rhipicephalus sanguineus* and in one out of 2 *Heamaphysalis elliptica* ticks (*G*. *Dobler*, *manuscript in preparation)*. All three *Rickettsia* species found are known to be pathogenic for humans, so although *R*. *africae* may be the predominant SFG pathogen, others could play a role.

However, SFG rickettsiae, transmitted by the brown dog tick *Rh*. *sanguineus*, like *R*. *massiliae* or *R*. *conorii*, could also be the cause of the observed antibody prevalence; but those would be expected to be more frequent in urban settings [29], which does not correspond to our findings on population density.

In Tanzanian agriculture, most cattle herding is done by male family members, thus the slightly higher seroprevalence in male participants may corresponds to cattle contact as a risk factor.

Our analysis may be limited by the fact that we did not take into account the “site” variable. In EMINI, sites were specifically chosen to represent different socio-economic and ecological variables, thus the variable “site” is clearly collinear with most variables tested in this analysis. Including the variable “site” into the analysis would have implied that these variables, e.g. elevation or population density would have been corrected for and their correlation with SFG IgG rendered insignificant. This would probably have been inadequate and have led to most likely false observations of no correlation between SFG IgG and those variables. However, hypothetical variables inherent to the “site” that we were not able to capture and that may have influenced the site’ s SFG IgG prevalence might thus make the association of SFG IgG to the variables analysed stronger than adequate.

In conclusion, our findings are compatible with previous descriptions and add information, especially on risk factors for SFG rickettsioses in southwestern Tanzania. SFG might thus contribute to disease burden—other studies describe SFG rickettsioses to be frequent in travellers from Africa, and as endemic diseases in African countries [4,5,12,30]. Future studies should aim to detect the pathogen in acute infection, and to describe the local transmission cycle in order to validate the identified risk factors in a prospective way.

## Supporting Information

S1 Checklist STROBE checklist. (DOC)
